# Contraception for Adolescents

**DOI:** 10.4274/jcrpe.galenos.2019.2019.S0003

**Published:** 2020-02-06

**Authors:** Nicole Todd, Amanda Black

**Affiliations:** 1 Department of Obstetrics and Gynecology, University of British Columbia, Vancouver, Canada; 2 Department of Obstetrics and Gynecology, University of Ottawa; and The Ottawa Hospital Research Institute, Ottawa, Canada

**Keywords:** Adolescent, contraception, family planning, long acting reversible contraception, counselling, contraceptive services, pregnancy in adolescence/prevention and control

## Abstract

Although pregnancy and abortion rates have declined in adolescents, unintended pregnancies remain unacceptably high in this age group. The use of highly effective methods of contraception is one of the pillars of unintended pregnancy prevention and requires a shared decision making process within a rights based framework. Adolescents are eligible to use any method of contraception and long-acting reversible contraceptives, which are “forgettable” and highly effective, may be particularly suited for many adolescents. Contraceptive methods may have additional non-contraceptive benefits that address other needs or concerns of the adolescent. Dual method use should be encouraged among adolescents for the prevention of both unintended pregnancies and sexually transmitted infections. Health care providers have an important role to play in ensuring that adolescents have access to high quality and non-judgmental reproductive health care services and contraceptive methods in adolescent-friendly settings that recognize the unique biopsychosocial needs of the adolescent.

## Introduction

Adolescents, defined by the World Health Organization (WHO) as individuals between the ages of 10-19 years (1), represent almost one-fifth of the world’s population. During adolescence, young people navigate numerous physical, cognitive, emotional, and behavioural changes as they acquire increasing autonomy and experiment in many areas. Experimentation may include alcohol or drug use, smoking, and sexual activity, all of which may be associated with sexual and reproductive health risks such as unintended pregnancy and sexually transmitted infections (STIs).

The United Nations and the WHO consider that access to safe, voluntary family planning is a human right because it is essential for promoting gender equality, advancing the autonomy of women, and reducing poverty (2,3). The WHO has identified key elements in quality of care in family planning which include: having choice among a wide range of methods; patient-provider relationships based on respect for informed choice, privacy, and confidentiality as well as the cultural and religious beliefs of the young woman; providing evidence-based information on the effectiveness, risks, and benefits of the different contraceptive methods; having technically competent trained health care workers; and having convenient access to a range of relevant services (2). The WHO also states that no method of contraception is contraindicated on the basis of age alone (4). These position statements extend to adolescents who also have the right to sexual and reproductive health services, including contraceptive care and counselling. However, access to contraceptive education and information and the availability and accessibility of contraceptive methods may be affected by the complex dynamics of social, cultural, political, and religious influences, particularly for adolescents.

## Sexual Behaviour and Unintended Pregnancy

In most Western countries, the median age of first intercourse is around 17 years. By age 18, 60% of females will have had sexual intercourse and by age 20 years almost 80%. Many have had more than one partner (5,6,7,8). Adolescents have the lowest level of contraceptive knowledge and use (9). Initiation of sexual activity while they lack adequate knowledge and skills to protect themselves places adolescents at higher risk of unwanted pregnancy, unsafe abortion, and STIs (10). Although there appears to be an increase in contraceptive use at first intercourse, many adolescents still do not use any method of contraception at first intercourse or do not continue to use contraception consistently (7,11). The most commonly used method of contraception at first intercourse is the male condom, which is important from the STI prevention perspective but is less reliable as a contraceptive method due to typical use failure rates that are significantly higher than those seen with other contraceptive methods (12).

Unintended pregnancy in adolescents can have major consequences for the young woman, her family, and society. The use of effective contraceptive methods is a cornerstone of adolescent pregnancy prevention. Although adolescent pregnancy rates are decreasing worldwide, adolescent mothers make up 11% of births (13). Although there are variations in cultural norms around age of marriage and childbearing, the majority of adolescent pregnancies are unintended (9,14,15). Adolescent pregnancy contributes to maternal and child mortality, with complications from pregnancy and childbirth being the leading cause of death for girls aged 15-19 years (13). Adolescents who give birth face significant socioeconomic challenges. Adolescents at greater risk of unintended pregnancy include those who are living in poverty, with low education and fewer employment opportunities, and marginalized populations. Pregnancy itself is an important opportunity to counsel on future contraceptive plans, as rapid repeat pregnancy is common among adolescent mothers (16). The Centers for Disease Control (CDC) Medical Eligibility Criteria for Contraceptive Use (MEC) provides guidance on post-partum contraceptive options (17).

## Barriers to Contraceptive Access and Use

Barriers to accessing contraceptive information and methods include social or culture taboos, legal restrictions, health care provider (HCP) attitudes, and healthcare systems (9,10). The acceptability and availability of contraception for adolescents varies by region and even by countries in the same region. Adolescents may experience barriers accessing contraception including inconvenient medical clinic hours, financial restrictions, lack of confidentiality, and lack of provider training. HCPs themselves may act as medical barriers by imposing their own personal values/moralistic beliefs on the adolescent, by applying inappropriate medical contraindications on recommendations for contraceptive use, by delaying initiation of contraception unnecessarily (i.e. waiting until the next menses or until STI screening results are available), by requiring unnecessary investigations prior to contraceptive initiation (i.e. by erroneously insisting on a Pap smear prior to starting contraception), or by perpetuating unfounded myths about contraceptive use (18). HCPs should ensure that they have the necessary skills and knowledge to provide unbiased, non-judgemental, evidenced-based, adolescent-friendly sexual health and reproductive health care and to be able to dispel common myths and misperceptions about contraceptive use (Table 1) (9).

The cost of contraception services and methods is a potential barrier for adolescents. Contraception may be prohibitively costly for an adolescent and the need for parental financial assistance may compromise confidentiality. Although contraception is provided at no cost in some countries, in other countries contraception is covered by private healthcare and/or by the patient paying directly. Provision of contraception at no cost may remove one financial barrier but does not guarantee high rates of utilization. Nonetheless, universal subsidies for contraception appear to be cost-effective (25). The annual direct cost estimates for unintended pregnancy are $320 million in Canada and $4.6 billion in the United States (26,27). Contraceptive non-adherence accounts for 69% of this cost. Cost models have shown that provision of/switching to long-acting reversible-contraceptives (LARC) would reduce contraceptive failures and lead to cost neutrality within 12 months (26,27). The Contraceptive CHOICE Project determined that provision of free contraceptives to adolescents reduces teen pregnancy, teen birth, and abortion (28) while yielding significant cost savings (29). The CHOICE Project also found that when cost is removed, the majority of adolescents (~70%) would choose LARC.

## Contraceptive Counselling

There should be no restrictions on the ability of adolescents to receive complete and confidential contraceptive services. An assurance of confidentiality will increase the willingness of adolescents to disclose sensitive health information and seek health care advice, while a loss of confidentiality can negatively impact an adolescent’s participation in sexual health services (30). Confidentiality, including its scope and limits, should be discussed with adolescents and caregivers, and reiterated once the adolescent is alone. Regrettably, adolescents’ legal rights to confidential family planning services vary by region and change over time (31). Adolescents should also be aware of instances where confidentiality may need to be breached (32). HCPs should consult local laws regarding confidentiality and age of consent, which may vary by region. An adolescent’s choice of contraception should be respected, and contraception should never be coercive.

The clinic should be welcoming to adolescents, ideally with flexible scheduling, convenient times (timed around school), and age appropriate visual aides (33). Scheduled follow-up visits are important to ensure method acceptability and ongoing contraceptive adherence.

HCPs should engage in a shared decision making process with adolescents. There are many suggested approaches to contraception counselling. The CDC suggest that sexual history taking should include the “5Ps”: Partners, Practices, Protection from STIs, Past history of STIs, and Pregnancy Prevention (33). This can help HCPs and adolescents work toward a contraceptive plan that is focussed on anticipatory guidance, education, and disease prevention. Another approach to contraception counselling is the “GATHER” approach where the HCP **G**reets and builds rapport, **A**sks questions and listens, **T**ells her relevant information to help her make an informed choice, **H**elps make a decision and provides other related information, **E**xplains the method in detail including its effectiveness, potential side effects, and how to use it, and lastly has the patient **R**eturn for advice or further questions (34). Another approach to contraception counselling can be found in Table 2. Adolescents should be asked about intimate partner violence, and specifically about reproductive coercion.

HCPs should counsel on all available contraceptive options without bias. Effectiveness, advantages and disadvantages should be discussed. Adolescents should be advised that failure rates are highest for user dependent methods (e.g. natural family planning, withdrawal, condoms, oral contraceptives) (12). LARC methods act continuously and are less user-dependent [e.g. contraceptive implants and intrauterine contraceptives (IUCs)]. A recent Cochrane review did not find significant differences amongst hormonal contraception, levonorgestrel releasing system (LNG-IUS), and copper intrauterine device (Cu-IUD), although the studies were small, and of low to moderate quality (35). Anticipatory discussion around anticipated menstrual side effects can reduce discontinuation of the shorter acting methods (36).

**Figure 1:**
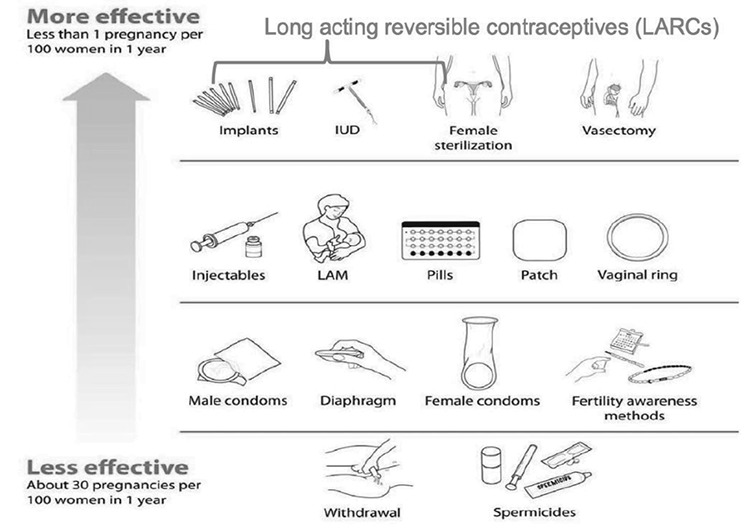
World Health Organization Tiered approach to contraceptive effectiveness *Adapted from Family Planning: A Global Handbook for Providers (2018 Update) (37) IUD: intrauterine device

The WHO has developed a tiered system to discuss contraception (Figure 1) (37):

**Tier 1:** LARC are methods that do not rely on the user.

**Tier 2:** Methods that rely on consistent use daily (pill), weekly (patch), every three weeks (vaginal ring), every three months depo-medroxyprogesterone acetate (DMPA).

**Tier 3:** Methods that rely on user during sexual activity (male and female condom, spermicide, natural family planning), or immediately after [emergency contraception (EC)].

Many international organizations have recommended moving to a tiered approach to contraceptive counselling, whereby HCPs present contraceptive options in order of contraceptive effectiveness and start the contraceptive discussion with Tier 1 LARC methods (8,33,38). Contraceptive effectiveness is one of a woman’s most important considerations when choosing a contraceptive method (39) and using top tier methods would achieve the highest effective contraception. However, while effectiveness is a paramount characteristic, it is important that tiered counselling focused on “LARC-first” does not become too directive or coercive, particularly in vulnerable populations (40). In a rights-based family planning framework, the choice of contraception should be made in collaboration with each individual adolescent taking into account safety, effectiveness, accessibility, and affordability while respecting her personal beliefs, culture, preferences, and ability to be adherent (25).

Age alone is not a contraindication to any contraceptive method (2,32,41). HCPs should address common myths and misperceptions (Table 1) as well as common side effects. Adolescents may fear weight gain, bleeding, acne, and mood side effects, while their parents may fear effects on future fertility and the risk of cancer. Regardless of the method of contraception chosen, adolescents should be counselled on the importance of the use of latex condoms to reduce the risk of STI acquisition (dual method) (25,38).

## Starting Contraception

Most contraceptive methods can be initiated at any time during the menstrual cycle provided that pregnancy or the possibility of pregnancy can be ruled out (Table 3) (41,42). The “Quick Start” method refers to starting a method immediately rather than waiting for the next menstrual period. Waiting to initiate contraception may place an adolescent at an increased risk of unintended pregnancy. Starting contraception immediately/at the time of the visit, has been associated with improved short-term compliance and is not associated with an increased incidence of breakthrough bleeding or other side effects (43,44). When the possibility of pregnancy is uncertain, the benefits of starting a combined hormonal contraceptive (CHC) (CHC: COC, vaginal contraceptive ring, contraceptive patch) likely exceed any risk. Thus CHC can be started immediately and a follow-up pregnancy test arranged in 2-4 weeks. Adolescents who choose to Quick Start contraception when a very early pregnancy cannot be completely excluded can be reassured that current evidence does not demonstrate an adverse impact of contraceptive hormone exposure on either fetal development or pregnancy outcomes (45,46). When using the Quick Start method, back-up contraception (barrier method and/or abstinence) should be used for the first seven consecutive days of contraceptive use unless it is initiated on the first day of menses (42). Adolescents may choose to start hormonal contraception on the first day of the next menstrual cycle or do a “Sunday start”. Starting on the first day of the menstrual cycle allows an adolescent to be reasonably sure that they are not pregnant. Initiating on a Sunday allows for a withdrawal bleed to occur on a Monday, assuming a seven-day hormone-free interval (HFI). CHCs, injectable progestins, or contraceptive implants may be started immediately after a surgical or medical pregnancy termination (47). An IUC can be inserted immediately after first or second trimester abortion.

In asymptomatic patients, there is no requirement for a pelvic exam prior to initiating contraception. Pap smear screening recommendations have changed in recent years and vary by region, but most no longer advocate for Pap smear screening in adolescents; some bodies recommend delaying screening until age 21 in sexually active women while others endorse delaying Pap smear screening until age 25. STI screening can be accomplished with urine sample for polymerase chain reaction, self-collection swabs, or cervical swab collection. STI screening is not a requirement prior to IUC placement. STI screening may be performed on the day of IUC insertion but insertion should not be delayed while waiting for the results, provided that there are no overt signs of infection. HCPs should provide at least a year-long prescription and should consider having samples on site to provide to adolescents (38). All adolescents should be counselled on how long to use back up contraception after starting a new contraceptive method. The Cu-IUD is effective immediately while CHC methods, the single rod implant, the LNG-IUS, and DMPA are effective after seven consecutive days of use. Additional information on what to do if they miss/delay taking their contraceptive method should be provided.

## Non-contraceptive Benefits

Counselling on contraceptive options should also include discussion about non-contraceptive benefits. Hormonal methods can provide improvement in heavy menstrual bleeding (HMB) and dysmenorrhea. CHC can also improve cycle regularity, acne, hirsutism, and premenstrual symptoms. Adolescents may prefer concealed options such as injectables, implants or IUC.

## Emergency Contraception

Regardless of the contraceptive method they choose, adolescents should be aware of EC and know that it can be used in the setting of contraceptive failure, such as condom interruption, non-adherence to hormonal contraception, or no contraceptive method used. HCPs should write prescriptions for EC, and provide information on how and when to access EC. Hormonal EC is available in many countries without a prescription. Increased availability of hormonal EC does not increase the frequency of unprotected intercourse (UPI), the likelihood of sexual risk-taking, or make women less likely to use effective contraception (48). Available EC options include: LNG-EC, 1.5 mg orally x 1 dose, high dose CHC (Yuzpe method), ulipristal acetate (UPA) (UPA-EC, 30 mg orally x 1 dose), mifepristone (low, mid dose) and insertion of Cu-IUD (25,49). The most effective EC is the Cu-IUD, which can be used up to seven days after UPI provided a pregnancy test is negative. It also has the additional benefit of ongoing contraception; however adolescents may experience barriers accessing a provider within the recommended time window (25,32). Hormonal EC can be offered up to 120 hours after UPI or contraceptive failure, although LNG-EC is more effective the sooner it is taken. UPA-EC may be used up to five days after UPI and may be more effective than LNG-EC in obese adolescents (50). There are no absolute contraindications to EC, aside from pregnancy or previous sensitivity reactions. Use of a Cu-IUD for EC has the same eligibility criteria as routine Cu-IUD insertion (2,41).

LNG-EC, UPA-EC, and mid dose mifepristone are all more effective than the Yuzpe method although all methods have been shown to decrease pregnancy rates (49). The Cu-IUD causes an inflammatory reaction that is toxic to oocytes, spermatozoa, and increases smooth muscle activity in fallopian tubes and myometrium preventing implantation. Hormonal EC works by impairing follicular development of the dominant follicle provided they are taken prior to ovulation. LNG-EC is preferred over the Yuzpe method owing to higher effectiveness - up to 85% if used within 72 hours. UPA-EC is more effective than LNG-EC likely due to its ability to disrupt ovulation even if taken after the LH surge has begun. For adolescents using LNG-EC or the Yuzpe regimen, hormonal contraception can be resumed immediately. In the case of UPA-EC, initiation of hormonal contraception should be delayed for five days due to potential interactions between the two medications that may affect effectiveness and UPA-EC’s ability to delay ovulation (51). Backup contraception and/or abstinence should be used until hormonal contraception has been taken for at least seven consecutive days. On the other hand, the Cu-IUD is immediately effective for ongoing contraception. EC users should have a pregnancy test if spontaneous menses do not occur within 21 days of EC use, if the next menstrual period is lighter than usual, or if it is associated with abdominal pain not typical of the woman’s usual dysmenorrhea. If a pregnancy occurs in a cycle during which oral EC was taken, the adolescent should be advised that there does not appear to be a harmful effect on pregnancy outcomes and there is no increased risk of congenital abnormality (48).

EC is a useful back-up method for condom use: if the condom breaks, slips, or is not used, there is still a further possibility of preventing pregnancy. However, the efficacy of hormonal EC is significantly lower than regular use of contraception and its preventive efficacy should not be overestimated. In most clinical scenarios, EC provision should be considered an opportunity for counselling and to start a continuous and effective contraceptive method as soon as possible (5). Quick Start is described previously.

## Medical Eligibility Criteria for Contraceptive Use in Adolescents

Although age itself is not a contraindication to the use of any method of contraception, reversible contraceptive methods are generally preferred in adolescents. Guidance for the safety of contraceptive use in women with certain characteristics or medical conditions are provided in the form of MEC from the WHO, the CDC, the Faculty of Sexual and Reproductive Healthcare, and other international organizations (4,17,52). For each medical condition/characteristic, contraceptive methods are placed in one of four categories to determine contraceptive eligibility (Table 4). The WHO and CDC also developed Selective Practice Recommendations for Contraceptive Use that recommend which tests and exams should be performed prior to providing contraception (2,41). Breast, pelvic and genital examination, Pap smears, and bloodwork are not recommended routinely because they do not contribute to increased safety of CHC use. Ideally, blood pressure and body mass index (BMI) should be recorded for adolescents prior to starting CHC but should not delay initiation of contraception. A medical history should be taken to alert HCPs to conditions or risk factors that might be a contraindication to contraceptive use.

## Contraceptive Options for Adolescents

### Intrauterine Contraception

IUCs are LARC methods that are highly effective and can be used by women of any age. Neither age nor nulliparity are contraindications to their use although rates of IUC expulsion are significantly higher in adolescents compared to older women regardless of parity or IUC type (4,53). Many international societies have stated that IUCs are a safe first line choice for adolescents (8,31,32,38,54,55) and encourage HCPs to counsel all adolescents on their use for the prevention of pregnancy due to their low typical use-failure rates and high one-year continuation rates. IUC rates have a 99% efficacy, with over 80% continuing with the method at one year (54). There are two types of IUCs: Cu-IUD and LNG-IUS. The Cu-IUDs may either have a frame (usually T-shaped) or be frameless and contain a varying amount of copper. The LNG-IUS’s (LNG-IUS 20, LNG-IUS 12, LNG-IUS 8) contain different amounts of levonorgestrel in their reservoir. The main mechanism of action of all IUCs is the prevention of fertilization.

Prior to providing or placing an IUC, absolute and relative contraindications should be reviewed. There is no requirement for pre-placement ultrasound. HCPs may require additional training for insertion. The success rate for insertion in adolescents is 96% (56). Adolescents may choose the LNG-IUS for its non-contraceptive benefits that include a reduction in menstrual bleeding and dysmenorrhea. The LNG-IUS 20 (Mirena^®^) is approved for treatment of HMB, and may prove beneficial for adolescents with HMB, bleeding disorders, and those on anti-coagulation (57). Although the LNG-IUS has less systemic absorption compared to CHCs, some adolescents experience hormonal side effects including acne, breast tenderness, headaches, and mood changes. Functional ovarian cysts may occur in LNG-IUS users, however these cysts are often asymptomatic and do not require further intervention (54). Adolescents choosing a Cu-IUD may be seeking a LARC method with minimal hormonal exposure. Cu-IUD users may experience increased menstrual blood loss and dysmenorrhea. Adolescents can be offered non-steroidal anti-inflammatory drugs (NSAIDs) and/or tranexaminic acid to help decrease menstrual blood loss and dysmenorrhea. With time, the number of unscheduled bleeding days tends to decrease with both LNG-IUS and Cu-IUD users. Occasionally IUC users may request IUC removal due to ongoing dysmenorrhea.

HCPs should counsel the adolescent about IUC insertion and not rush. Handouts may be helpful and can include information about the need for ongoing condom use to protect against STIs, duration of back-up contraception after insertion (seven days for the LNG-IUS, none required for Cu-IUD), recommendations for prophylactic NSAIDs for insertion, common initial side effects such as cramping or unscheduled bleeding, and when to seek medical assessment. Pre-placement NSAIDs have been shown to reduce discomfort post-insertion. Currently, there is no evidence to support routine pre- and post-placement ultrasound. Although in selected cases vaginal and/or oral misoprostol taken pre-procedure may help with IUC insertion, its routine use should be discouraged due to an increase in side effects such as bleeding, abdominal pain and cramping, fever, and higher pain scores post-IUC insertion (58). Paracervical blocks may reduce pain with tenaculum placement, but have not been shown to reduce pain with IUC insertion. Smaller diameter LNG-IUS’s (LNG-IUS 12, LNG-IUS 8) and Cu-IUDs may be associated with less pain on insertion. Adolescents should be offered IUC placement in the clinician’s office, and routine insertion in the operating room should be avoided unless this is the adolescent’s preference. Prior to IUC placement, the HCP should rule out the possibility of pregnancy (Table 3).

IUCs are not associated with an increased risk of pelvic inflammatory disease or STI acquisition although there is a small increased risk of pelvic infection seen within 21 days of IUC placement (59). STI screening should be performed in women at high risk of STIs prior to or at the time of insertion but it is not necessary to delay IUC insertion until the results are available. Positive results can be treated while the IUC remains *in situ* (54). Routine antibiotic prophylaxis at the time of IUC placement is not recommended. IUCs can safely be used in adolescents with a history of STI, including human immunodeficiency virus (HIV), although insertion should be delayed if there is evidence of mucopurulent discharge. Immunosuppression is not a contraindication to IUC use (4,8).

IUCs may be safely inserted in the immediate post-abortion and post-partum period (delivery to 48 hours). While there may be a slightly higher expulsion rate (10%), this should not be a barrier to offering placement. Immediate post-placental insertion should not be offered in the setting of chorioamnionitis and/or post-partum hemorrhage.

## Progestin-only Contraceptive Options

Progestin-only contraceptives do not contain estrogen and thus may be good options for young women who cannot take estrogen. There are few contraindications to progestin-only methods: current breast cancer (Category 4), breast cancer remission within five years, severe cirrhosis, hepatocellular adenoma, malignant liver tumour, and unexplained vaginal bleeding (Category 3) (4,17,60). Non-contraceptive benefits of progestin-only options include decreased dysmenorrhea and endometriosis-related pain. The most common side effect is unscheduled bleeding. All progestin-only contraceptive options are safe for adolescents, with the implant being a WHO Tier 1 contraceptive method (37).

### A) Contraceptive Implant

The single rod implant containing etonogestrel, an active metabolite of desogestrel, is the most effective method of reversible contraception with an efficacy of 99%. It is effective *in situ* for up to three years, although it is likely effective for up to four years, and high continuation rates are seen at one and two years (28,60,61). Its contraceptive effect is due to cervical mucous thickening, thinning of endometrial lining, and ovulation inhibition. The most common side effect is unscheduled bleeding which is variable and does not necessarily improve with time. Implant users requesting removal often cite abnormal uterine bleeding, weight gain, or acne as the reason for removal (62). Functional cysts can be seen in users, but usually do not require further intervention (60). The implant does not have an adverse effect on bone mineral density (BMD) such as that seen with DMPA, likely owing to ongoing ovarian activity that allows for endogenous estradiol to support bone health, but there is limited evidence in adolescents. This Tier 1 method may be a good option for adolescents because it is non-coitally dependent, does not require daily user action, and is discrete. Advantages of this LARC include 3-year duration of effectiveness, reversibility, discretion, and can be used by adolescents who have contraindications to estrogen. It can be seen on X-ray. Contraceptive implants can be inserted post-abortion, and immediately post-partum thereby reducing rapid repeat pregnancy and repeat abortions among adolescents (63).

### B) DMPA

DMPA-IM is an intramuscular injection that is administered every 12 weeks by a HCP. A lower dose subcutaneous version (DMPA-SC) that can be self-administered is available in some countries. DMPA inhibits pituitary gonadotropins, leading to anovulation and causes thickening of cervical mucous. Advantages of this method include discretion, infrequent dosing, and non-contraceptive benefits such as reductions in dysmenorrhea, premenstrual symptoms, HMB, fibroids, anemia, seizures, and sickle cell crises (8,60). It is one of the few systemic hormonal contraceptives that can be reliably used with liver-enzyme inducing drugs because its concentrations are not affected (5). Disadvantages may include having to access a HCP for intramuscular injections, unscheduled bleeding, delayed return to fertility, and weight gain. Adolescents using DMPA appear to gain more weight than non-users or users of other contraceptive methods (64). Adolescents who experience more than a 5% weight gain after six months of DMPA use may be at risk of continued excessive weight gain (65). DMPA has high rates of amenorrhea, with up to 68% of DMPA users being amenorrheic at 24 months. Although unscheduled bleeding may decrease in amount and frequency with time, irregular bleeding is a common reason for discontinuation.

DMPA use can be associated with a reversible BMD loss, likely due to the estrogen deficiency that accompanies its use (66). This may be of concern in adolescence, when bone accrual should be occurring (67,68). The BMD loss associated with DMPA use is greatest in the first one to two years which has led several organizations to recommend a maximum duration of use of two years. The bone loss seen with DMPA use is similar to bone loss seen with pregnancy and appears to return to baseline within two years of discontinuation (69,70). Both the American College of Obstetricians and Gynecologists and the Society of Obstetricians and Gynaecologists of Canada have recognized the risks of unintended pregnancy in adolescents if their contraceptive options are limited and hence have stated that there should no restriction on the use of DMPA or duration of use in women who are otherwise able to use the method (60,71). The WHO has determined that for females younger than 18 years, the advantages of using DMPA generally outweigh the theoretic safety concerns regarding fracture risk (72).

Routine BMD monitoring is not recommended in adolescents using DMPA because dual energy X-ray absorbtiometry has not been validated in these populations. Although studies have demonstrated that low dose estrogen supplementation limits BMD loss in adolescent DMPA users, it isn’t recommend because of potential adverse effects and because there is lack of clinical evidence for the prevention of fractures in the adolescent population (71). Adolescent DMPA-users should be counselled on adequate calcium and vitamin D, weight bearing activity, and avoidance of alcohol, caffeine, and smoking which can be associated with BMD loss. HCPs should discuss the overall risks and benefits with DMPA users at regular intervals.

Recently, the WHO reviewed concerns about potential increased HIV acquisition in DMPA users. They determined that for women at high risk of HIV acquisition there are no restrictions for use of reversible methods (73). A recent randomized controlled trial did not find an increased risk of HIV acquisition amongst Cu-IUD, DMPA-IM, or LNG implant users (74).

### C) The Progestin-only Pill (POP)

The POP is taken every day, without a HFI. This method works via thickening cervical mucous with anovulation seen in only 50% of user. Adolescents should be counselled that POP needs to be taken at the same time every day to avoid pregnancy risk. It is often used as post-partum contraception when women are breastfeeding. Users may continue to have regular cycles, however, unscheduled bleeding is the most common reason for discontinuation

## Combined Hormonal Contraception

CHC methods contain an estrogen and a progestin. They include the pill, patch, and vaginal ring. In the absence of medical contraindications adolescents can safely use CHC. Absolute and relative contraindications should be reviewed prior to initiation (4,17). Common side effects including unscheduled bleeding, nausea, and headaches, should be discussed with the adolescent prior to initiation, as this improves continuation (36). Adolescents and young women can be counselled that they can take the CHC with a 4- or 7-day HFI, and/or can take cyclically or in extended cycle (Skipping periods). Benefits of extended cycle use include reduction in dysmenorrhea, HMB, acne, anemia, and conditions exacerbated by cyclic variations (e.g. migraine without aura, epilepsy, irritable bowel syndrome, inflammatory bowel disease, mood, behaviour) (8,75). Women taking CHC in extended cycle either experience equivalent or less unscheduled bleeding compared to cyclic counterparts (75). Extended/continuous cycles can be achieved by using the hormone for two, three, or more cycles back-to-back, without taking a HFI and having a withdrawal bleed. The safety of this approach is well established and adolescents should be counselled that not experiencing bleeding during a HFI is safe, as evidenced by equivalent endometrial assessment via ultrasound and/or endometrial biopsy (75). For contraceptive efficacy, a HFI should not be taken until at least 21 consecutive days of hormonal contraception has been used. It is helpful to provide adolescents with written instructions or website links on how to take CHC in extended cycle, and what to do if a dosage is missed. Follow-up should be scheduled at one and three months to ensure the method is acceptable and to assess side effects.

**A. Combined Oral Contraceptive (COC) pills** are the most popular hormonal contraceptives among adolescents. Typical use failure rate is 9% (12) and is usually secondary to non-adherence. Adolescents should be counselled on behaviours to increase contraceptive adherence including: regular schedule, phone alarm, and family member support (8,9). Adolescents should be provided with resources (paper, app, online) to assist when pills are missed.

**B. The Contraceptive Patch** should be placed on the buttocks, upper arm, upper torso, or abdomen once weekly for three weeks. During the HFI in the fourth week, a withdrawal bleed usually occurs. In obese adolescents, there may be a slightly higher risk of failure with the patch (76) but obesity is not a contraindication to use of the contraceptive patch (4,17). It can be used continuously for menstrual suppression if desired.

**C. The Vaginal Contraceptive Ring** is inserted into the vagina by the adolescent and should remain in the vagina for three weeks (21 days), although pharmacokinetic data indicate that it is effective for at least 28 days (77). When the ring is removed, the adolescent can choose to have a 4- to 7-day HFI or she can insert a new ring immediately to avoid having a withdrawal bleed. At no time should the HFI exceed seven days. The ring can stay in the vagina during sexual intercourse but if the adolescent does wish to remove it during intercourse, it should not remain out of the vagina for more than three hours (42).

## Considerations with Combined Hormonal Contraceptive

**i. Weight gain:** A Cochrane review did not find a significant association between COC or transdermal CHC and weight gain (78). There is currently insufficient evidence to link CHC use with weight gain. When counselling adolescents about weight gain, it is important to discuss ongoing physical development, and average weight changes for women over a year.

**ii. Mood: **Data on CHC effect on mood is conflicting. Placebo-controlled trials have not demonstrated a significantly increased risk of mood changes in CHC users compared with placebo users, and there is some evidence that COCs are protective for mood (79). COC’s containing drosperinone are associated with an improvement in premenstrual dysphoric disorder symptoms (80). Conversely, a large Danish prospective cohort study found an increased risk for first use of an antidepressant and first diagnosis of depression among users of different types of hormonal contraception, with the highest rates among adolescents (81). HCPs should counsel adolescents that CHC may be associated with mood changes, but there is no conclusive evidence linking CHC to depression (32).

**iii. Venous thromboembolism (VTE):** The baseline risk of VTE in adolescents is very low (1 per 10,000). CHC use is associated with a 3-fold increase risk for VTE with an absolute risk of 3-4 per 10,000 in adolescents. There currently is inadequate data to support preferential prescribing related to increased VTE risk based on type of progestin or dose of ethinyl estradiol (82). Prospective cohort studies do not seem to show a significant difference in VTE risk by progestin type (83,84). Routine thrombophilia screening in adolescents prior to initiating CHC is not advised.

**iv. BMD:** Adolescence is a time of bone mass accrual which continues up to approximately age 25 years (38). Although data on CHC effects on BMD is conflicting, there is currently no evidence supporting increased risks of osteoporosis or fracture in CHC users (72,85). Early data has suggested that in healthy adolescents, COCs with at least 30 mcg ethinyl estradiol may be preferred due to poorer bone mineralization seen with lower dose options (38), and that extended regimens may be preferred to 28-day cyclic regimens because there is greater bone accrual (86). Adolescents with eating disorders are at risk for decreased BMD. Although a recent study suggested COC use was associated with normalization of bone resorption markers in adolescents with anorexia nervosa and may limit bone loss (87), CHCs are generally not recommended for prevention of osteoporosis in this population (32).

**v. Obesity:** There are no contraindications to CHC use based on body weight and/or BMI alone (17,42). Studies demonstrate either equivalent or increased pregnancy rates among obese CHC users, however more high quality studies are needed (88).

## Barrier Contraception

Male condoms are the most commonly used contraceptive method at first intercourse, and one of the most commonly used methods among adolescents (9). This method retains its popularity due to its low costs and lack of need for a prescription. Typical use failure rates are as high as 18% and may be higher in adolescents due to inconsistent/incorrect use (8,89). HCPs can help ensure that adolescents understand proper condom use including sizing, placement, storage, and safe lubricants as well as how to negotiate condom use with their partners (32,89). There are concerns that adolescents choosing LARCs have the lowest rates of dual method use (90). Regardless of the contraceptive method chosen, HCPs should encourage adolescents to continue to use condoms for STI prevention as well as contraceptive back-up in the event of a contraceptive failure and/or non-use.

## Conclusion

The ability to freely choose when and how many children to have is a basic human right. Contraception is an important pillar for the prevention of unintended pregnancy in adolescents. HCPs should strive to provide care within the human rights based framework and to work with adolescents to find a method that best meets their personal biopsychosocial needs and that they will be able to adhere to. Adolescents should have access to a wide range of contraceptive options with LARCs being first line options due to their greater effectiveness. However, as LARC uptake increases among adolescents, it is important to incorporate messages about condom use specifically for STI prevention. Healthcare providers must provide counselling that is appropriate to the adolescent, acknowledges how they access health care, and is not perceived as directive or coercive.

## Figures and Tables

**Table 1:**
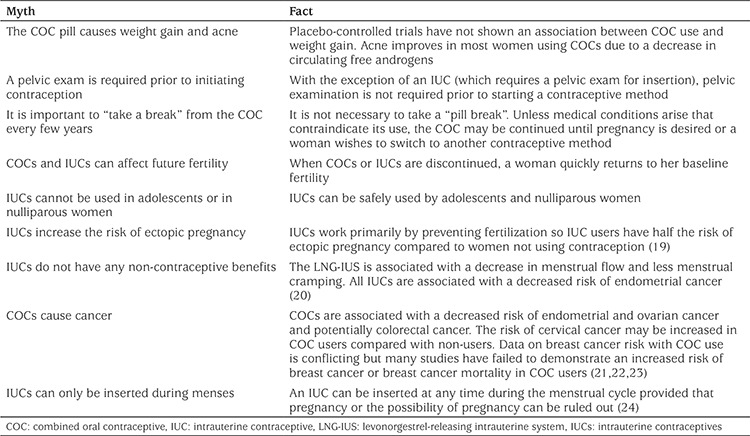
Contraceptive myths and misperceptions

**Table 2:**
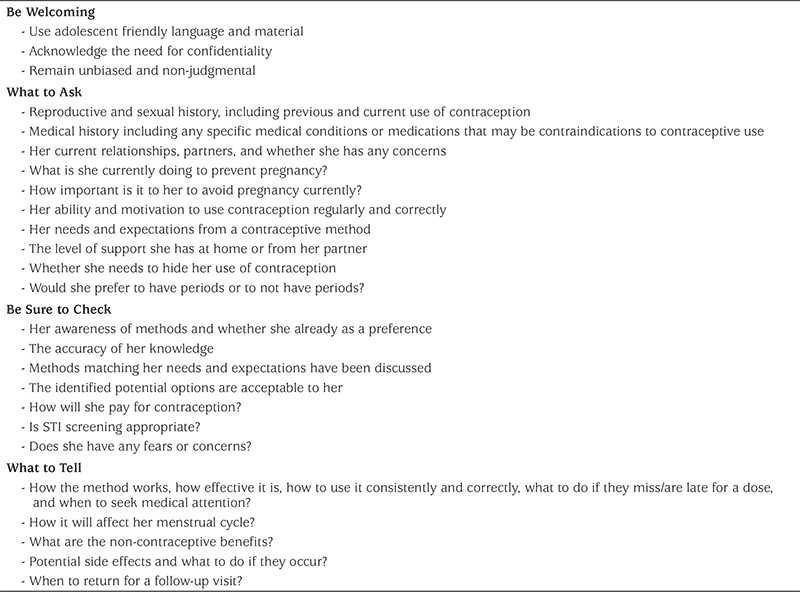
Contraceptive counselling in the adolescent

**Table 3:**
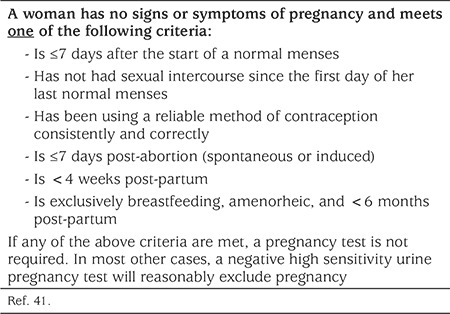
Criteria for being reasonably certain a woman is not pregnant

**Table 4:**
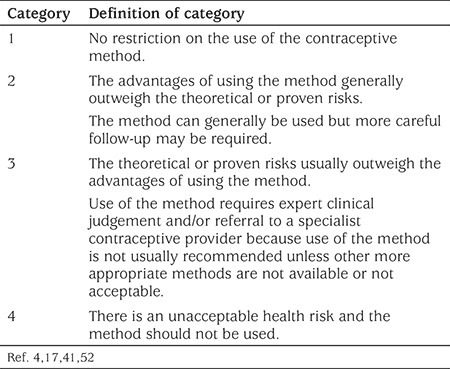
Medical Eligibility Criteria categories for contraceptive use
